# Coxsackievirus A16 utilizes cell surface heparan sulfate glycosaminoglycans as its attachment receptor

**DOI:** 10.1038/emi.2017.55

**Published:** 2017-07-26

**Authors:** Xueyang Zhang, Jinping Shi, Xiaohua Ye, Zhiqiang Ku, Chao Zhang, Qingwei Liu, Zhong Huang

**Affiliations:** 1 Unit of Vaccinology & Antiviral Strategies, CAS Key Laboratory of Molecular Virology & Immunology, Institut Pasteur of Shanghai, Chinese Academy of Sciences, Shanghai 200031, China

**Keywords:** attachment, coxsackievirus A16, heparan sulfate glycosaminoglycan, receptor

## Abstract

Coxsackievirus A16 (CVA16) is one of the major pathogens responsible for hand, foot and mouth disease, which affects more than two million children in the Asian-Pacific region annually. Previous studies have shown that scavenger receptor B2 is a functional receptor for CVA16 that facilitates the uncoating process. However, it remains unclear whether other receptors are required for efficient CVA16 infection. In this study, by using a variety of assays we demonstrated that CVA16 utilizes surface heparan sulfate glycosaminoglycans as its attachment receptor. We further showed that five surface-exposed positively charged residues located in a cluster at the five-fold vertex of the virion are critical to heparan sulfate binding and cellular attachment of CVA16. Among the five residues, the arginine at position 166 (R166) of VP1 capsid protein appeared to be the most important for the interaction between CVA16 and heparan sulfate. Alanine substitution at this site (R166A) almost completely abolished heparan sulfate binding and cellular attachment of the virus. Our work achieves insight into the early events of CVA16 infection, thereby providing information that may facilitate the rational design of antiviral drugs and vaccines against CVA16 infection.

## INTRODUCTION

Coxsackievirus A16 (CVA16) is one of the major pathogens responsible for hand, foot and mouth disease in infants and young children.^1^ A proportion of hand, foot and mouth disease patients infected with CVA16 may present with encephalitis, myocarditis and pneumonitis, which in some cases can be fatal.^2, 3, 4, 5^ Efforts have been made toward the development of CVA16 vaccines.^1, 6, 7, 8, 9, 10^

CVA16 is a non-enveloped virus belonging to the *Enterovirus* genus of the *Picornaviridae* family.^11, 12^ The virus contains a single-stranded positive-sense RNA genome ~7.4 kb in length,^13^ which is encapsidated in a spherical protein shell that consists of 60 copies each of VP1, VP2, VP3 and VP4 subunit proteins.^14, 15^ Recent studies have identified human scavenger receptor B2 (SCARB2) as an uncoating receptor for both EV71 and CVA16.^16, 17^ However, another EV71 receptor, human P-selectin glycoprotein ligand-1, which facilitates EV71 infection in Jurkat T cells, does not support CVA16 infection of the same cell line.^18^ It remains unknown whether other co-receptors are required for efficient CVA16 infection *in vitro* and *in vivo*.

Attachment to susceptible cells is the first step of the viral entry process. Previous studies have shown that the initial binding of a number of enveloped and non-enveloped viruses to cells is facilitated by glycosaminoglycans (GAGs) on the cell surface. Specifically, it has been reported that heparan sulfate-specific GAGs serve as an attachment receptor for several picornaviruses, including echovirus,^19^ human rhinovirus,^20, 21^ enterovirus 71,^22, 23^ coxsackieviruses B3 and A9^24, 25^ and foot-and-mouth disease virus.^26^ However, it remains unexplored whether heparan sulfate also mediates the attachment/entry of CVA16. Therefore, in the present study, we comprehensively investigated the role of heparan sulfate-specific GAGs in CVA16 infection of susceptible cells. Our results demonstrated that cell surface heparan sulfate serves as an attachment receptor for CVA16. Moreover, five surface-exposed positively charged residues on the VP1 subunit were found to be critical for heparan sulfate binding and cellular attachment of CVA16.

## MATERIALS AND METHODS

### Cells and viruses

Human rhabdomyosarcoma (RD, Cat# CCL-136) and Vero cells (Cat# CRL-1586) were obtained from the American Type Culture Collection (ATCC, Manassas, VA, USA) and grown as previously described.^27^ CVA16 strains SZ05 and G08 were described in a previous study.^7^ The viruses were propagated in Vero cells. Virus titers were determined in Vero cells as previously described^27, 28^ and expressed as 50% tissue culture infectious dose (TCID_50_).

### Western blot assay

Western blot analyses were performed as previously described^27^ with a polyclonal antibody against CVA16 VP1 protein as the detection antibody.

### Quantitative real-time RT-PCR

RNA was extracted and reverse transcribed to produce cDNA as previously described.^13^ The resultant first-strand cDNA was used as a template for quantitative real-time PCR (qRT-PCR) analysis. The qRT-PCR assay was performed using a SYBR Premix Ex Taq kit (TaKaRa, Dalian, China) with an Applied Biosystems 7900HT real-time PCR system. The CVA16-specific primers used were CVA16-RT-F (5′-ATC CAG TAA GGA TCC CAG ACT-3′) and CVA16-RT-R (5′-GAT TTG CAT AGT GGA GAG CAG-3′). β-actin mRNA was also measured, serving as an internal control, with a pair of primers: β-actin-RT-F (5′-GGA CTT CGA GCA AGA GAT GG-3′) and β-actin-RT-R (5′-AGC ACT GTG TTG GCG TAC AG-3′). Data analysis was performed using the 2^−ΔΔCT^ method as previously described.^29^

### Inhibition of CVA16 infection with soluble heparin

For the infection inhibition assay, CVA16 (100 TCID_50_ in a volume of 50 μL) was mixed with an equal volume of fourfold serially diluted heparin sodium salt (Sanjie, Shanghai, China) and then incubated at 37 °C for 1 h. The mixture was added to RD cells pre-seeded in a 96-well plate, followed by incubation at 37 °C. Three days later, the cell medium was measured for cell viability using a methylthiazolyldiphenyl-tetrazolium bromide (MTT)-based method as previously described.^30^ After color development, absorbance was determined at 490 nm in a 96-well plate reader. For a given sample, the relative viability was calculated by normalization of its *OD*_490_ nm value against that of the virus-only sample as follows: relative viability (%) = (*OD*_490_ nm of the given sample - *OD*_490_ nm of the virus-only sample)/(*OD*_490_ nm of the cell-only sample - *OD*_490_ nm of the virus-only sample) × 100.

### Inhibition of CVA16 attachment with soluble heparin

For an attachment inhibition assay, diluted GAGs were mixed with CVA16 virus (300 TCID_50_) and incubated at 37 °C for 1 h. Then, the mixture was added to 1 × 10^5^ RD cells pre-seeded 1 day ahead in a 24-well plate and incubated at 4 °C for 2 h. After the incubation, the cells were washed with serum-free medium three times, and the cell-bound virus was quantified by SYBR quantitative real-time PCR as described above.

### Enzymatic removal of heparan sulfate from the surface of RD cells

Heparinase I (Cat# H2519, Sigma, St Louis, MO, USA) was reconstituted in digestion buffer (PBS containing 0.5 mM MgCl_2_, 0.9 mM CaCl_2_ and 0.1% BSA). Various concentrations (1, 2.5, 5, 10 mIU/mL) of heparinase I in a final volume of 200 μL were added to 6 × 10^4^ RD cells and incubated at 37 °C for 1 h. The cells were then washed with the digestion buffer and subsequently incubated with CVA16/SZ05 or CVA16/G08 (8.6 × 10^3^ TCID_50_) at 4 °C for 1 h. After the incubation, the cells were washed twice with serum-free medium. Cell-associated virus was quantified by SYBR quantitative real-time PCR as described above.

### Treatment with sodium chlorate

RD cells were grown in a 24-well plate in medium containing 6.25, 12.5, 25 or 50 mM sodium chlorate (Sigma) for 40 h. Then, the cells were infected with CVA16 (100 TCID_50_) and incubated at 4 °C for 2 h. After three washes, the cells were analyzed for viral titer by quantitative real-time PCR as described above.

### Pull-down assay

For pull-down of wild-type CVA16, different amounts (5 × 10^5^ or 3 × 10^6^ TCID_50_) of CVA16/SZ05 or CVA16/G08 were mixed with 20 μL of heparin–agarose beads (Sigma) or 20 μL of empty agarose beads (AOGMA, Shanghai, China). The mixture was incubated at 4 °C for 2 h with gentle rotation in a rolling mixer. After incubation, the mixture was centrifuged at 2000 r/min for 5 min. The resulting precipitate was washed with PBS three times and then subjected to western blotting with an anti-CVA16 VP1 polyclonal antibody as described above.

For pull-down of CVA16 mutants, viruses (4 × 10^6^ RNA copies) in a final volume 500 μL were mixed with 20 μL of heparin–agarose beads in RNase-free tubes, followed by incubation at 4 °C overnight. After centrifugation at 2000 r/min for 5 min, the supernatant was removed. The precipitate was washed with PBS three times and then subjected to RNA extraction and quantitative real-time PCR as described above.

### Computer modeling

The crystal structure of CVA16 mature virus (PDB code: 5C4W)^14^ was obtained from the Protein Data Bank (PDB) (http://www.rcsb.org/pdb). The three-dimensional crystal structure of CVA16 pentamer was built using Swiss-PdbViewer software.

### Generation of CVA16 mutants

To determine the possible heparan sulfate-binding sites on CVA16 particles, we designed three CVA16 mutants with one or three of the five positively charged residues (Lys141, Arg166, Lys241, Lys242 and His245) of VP1 changed to alanine. These five positively charged residues were selected for mutagenesis study because they are highly exposed on the surface of the CVA16 capsid and therefore may potentially interact with negatively charged cell surface heparan sulfate. Accordingly, three mutant infectious clone plasmids, namely, pMD19-T7-141A-polyA, pMD19-T7-166A-polyA and pMD19-T-241/242/245A3-polyA, were generated using a previously established wild-type CVA16 infectious cDNA clone pMD19-CV^13^ as the backbone vector. The resulting plasmids were digested with *Not*I, purified and used as the template for *in vitro* transcription. *In vitro* transcription was performed using the Riboprobe system-T7 *in vitro* transcription kit (Promega, Madison, WI, USA) according to the manufacturer’s instructions. Then, the resulting RNAs were purified using the RNA Cleanup Kit (CWBIO, Beijing, China). Two micrograms of purified RNA derived from the mutant or wild-type constructs was individually transfected into 8 × 10^5^ pre-seeded Vero cells using Lipofectamine 2000 transfection reagent (Invitrogen, Carlsbad, CA, USA). At different time points post transfection, the cells and medium were collected and subjected to three freeze-thaw cycles. The presence of rescued viruses in the lysate was verified by RT-PCR and sequencing. Rescued mutant and wild-type viruses were further quantified based on RNA genome copy number determined by qRT-PCR as described above with the plasmid pIExBac-(CA16)3CD^31^ serving as the reference standard in the assay.

### Statistical analysis

All statistical analyses were performed using GraphPad Prism version 5. Statistical significance was determined by Student’s two-tailed *t*-test.

## RESULTS

### Soluble heparin inhibits CVA16 attachment and infection

**Figure 1:**
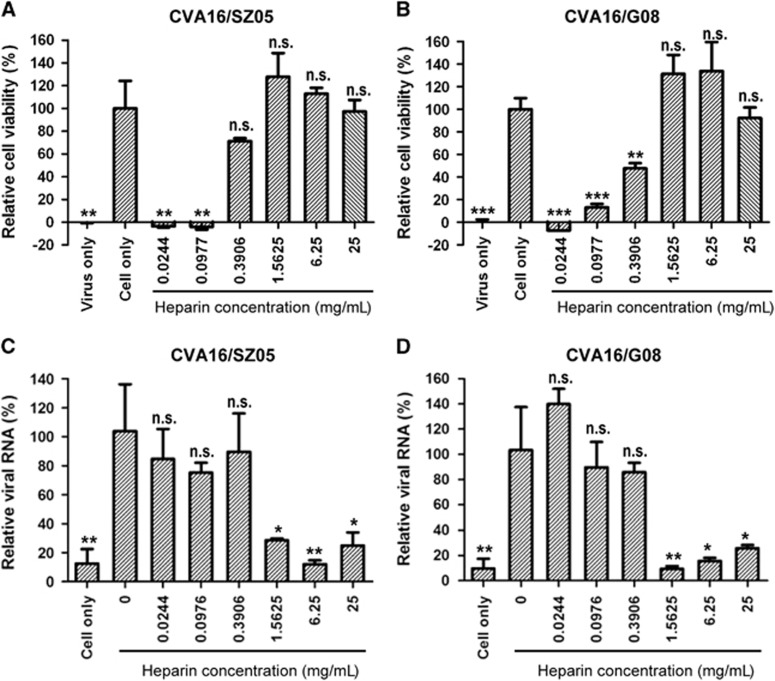
Inhibitory effect of soluble heparin on CVA16 attachment and infectivity. (**A**, **B**) Inhibition of CVA16 infection by soluble heparin. One hundred TCID_50_ of (**A**) CVA16/SZ05 or (**B**) CVA16/G08 was pre-incubated with various concentrations of heparin sodium salt for 1 h at 37 °C before proceeding to infection of RD cells at 37 °C. Three days later, the infected cells were analyzed for viability by a MTT assay. The data are reported as the mean±sd of relative cell viability for triplicate samples. Statistical significance between the treated samples and the control (cell-only) is indicated as follows: NS, *P*≥0.05; *P<0.05; **P<0.01; ***P<0.001. (**C**, **D**) Inhibition of CVA16 attachment to RD cells by soluble heparin. Three hundred TCID_50_ of (**C**) CVA16/SZ05 or (**D**) CVA16/G08 was mixed with various concentrations of heparin sodium salt at 37 °C for 1 h. Then, the mixture was added to 1 × 10^5^ RD cells pre-seeded 1 day ahead in a 24-well plate and incubated at 4 °C for 2 h. After the incubation, the cells were washed with serum-free medium three times. The cell-attached virus was quantified by qRT-PCR. The *y* axis shows the viral genomic RNA level of heparin-treated cells relative to that of the control (cells only infected with the virus). The mean±sd of triplicate wells are shown. The data are representative results of three independent experiments. Statistical significance between the treated samples and the control (cells only infected with the virus) is indicated as follows: NS, *P*≥0.05; *P<0.05; **P<0.01.

To investigate the role of heparan sulfate-specific GAGs in CVA16 infection, we first examined the effect of pretreatment with soluble heparin sodium salt on the infectivity of CVA16 in RD cells. As shown in Figure 1A, pretreatment with high concentrations (≥0.391 mg/mL) of heparin sodium salt significantly protected cells from CVA16/SZ05 infection-induced cytopathic effect as indicated by >50% cell viability compared to the mock-infected control; in contrast, lower concentrations (≤0.098 mg/mL) of heparin sodium salt exhibited no protective effect. Similar results were obtained when CVA16/G08, a low-passage strain, was used as the inoculum (Figure 1B). These data indicate that soluble heparin can inhibit CVA16 infection in a virus strain-independent manner.

We then determined whether heparin pretreatment affects the attachment step of CVA16 entry into RD cells. We found that heparin at concentrations of ≥1.5625 mg/mL could inhibit cellular attachment of both CVA16/SZ05 and CVA16/G08 (Figures 1C and 1D). Lower concentrations (≤0.3906 mg/mL) of heparin did not display an inhibitory effect on CVA16 attachment. Together, the above results indicated that high levels of heparan sulfate can block CVA16 attachment and infection.

### Enzymatic removal of surface heparan sulfate from RD cells weakens CVA16 binding

**Figure 2:**
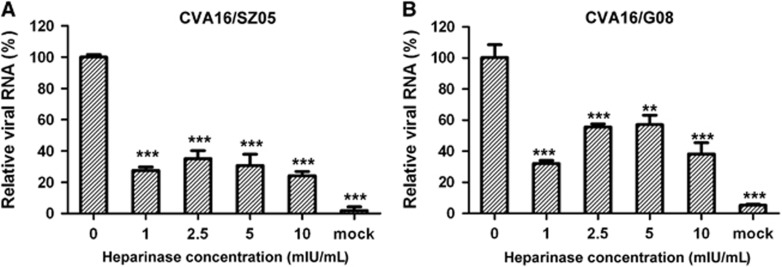
Heparinase I treatment impaired CVA16 attachment onto RD cells. RD cells were pretreated with heparinase I at 37 °C for 1 h and then inoculated with 8.6 × 10^3^ TCID_50_ of (**A**) CVA16/SZ05 or (**B**) CVA16/G08. After washes, the cells were analyzed for bound virus by qRT-PCR. The data are expressed as the mean±sd for triplicate samples. Statistical significance between the treated samples and the control (without heparinase I treatment) was determined by Student’s two-tailed *t*-test and is indicated as follows: **P<0.01; ***P<0.001. Representative results from two independent experiments are shown.

To determine whether surface heparan sulfate is required for CVA16 attachment to cells, we treated RD cells with heparinase I, which specifically cleaves heparin and highly sulfated domains in heparan sulfate, and then examined the treated cells for their ability to support CVA16 binding. As shown in Figure 2A, treatment with the enzyme at concentration as low as 1 mIU/mL reduced the amount of cell-bound CVA16/SZ05 by ~70% however, increasing the heparinase I concentration within the range of 1–10 mIU/mL did not significantly increase the attachment inhibition, suggesting that the concentration of 1 mIU/mL (the lowest concentration tested) might be above the threshold. A similar observation was recorded for the CVA16/G08 strain (Figure 2B), suggesting that the inhibitory effect of heparinase I treatment on CVA16 attachment is independent of the virus strain. These data demonstrate that surface heparan sulfate is required for efficient cellular attachment of CVA16.

### Interference with heparan sulfate biosynthesis inhibits CVA16 attachment and infection

**Figure 3:**
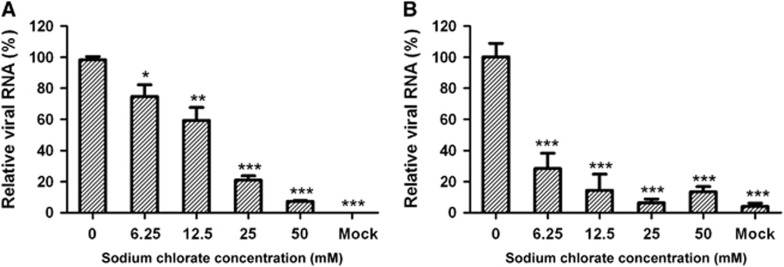
Sodium chlorate pretreatment of RD cells inhibited CVA16 attachment and infection. RD cells (1 × 10^5^/well) were cultured for 40 h in the presence of sodium chlorate at concentrations ranging from 0 to 50 mM. The treated cells were inoculated with 100 TCID_50_/well of CVA16/SZ05, followed by incubation (**A**) at 37 °C for 10 h or (**B**) at 4 °C for 2 h. The infected cells were analyzed for viral RNA by qRT-PCR. For each treatment, the viral RNA levels relative to those for the group infected with virus only (no sodium chlorate) are presented. The mean±sd for triplicate wells are shown. Statistical significance is indicated as follows: *P<0.05; **P<0.01; ***P<0.001.

Sodium chlorate can inhibit adenosine triphosphate sulfurylase, which is a crucial enzyme in heparan sulfate proteoglycan biosynthesis, resulting in significantly reduced sulfation of heparan sulfate.^32^ To determine whether the degree of sulfation affects heparan sulfate-mediated CVA16 binding and infection of target cells, we treated RD cells with various concentrations of sodium chlorate prior to CVA16 inoculation. Sodium chlorate treatment was found to significantly reduce CVA16 infection in a dose-dependent manner (Figure 3A). In the presence of 50 mM sodium chlorate, CVA16 infection was inhibited by 93% compared to the control (without sodium chlorate). We further determined the amount of cell-bound virus in pretreated and untreated samples by qRT-PCR. As shown in Figure 3B, the amount of CVA16 associated with the target cells was significantly reduced upon sodium chlorate treatment. These results demonstrate that the degree of sulfation is important for heparan sulfate to support CVA16 attachment and infection.

### CVA16 directly binds heparin

**Figure 4:**
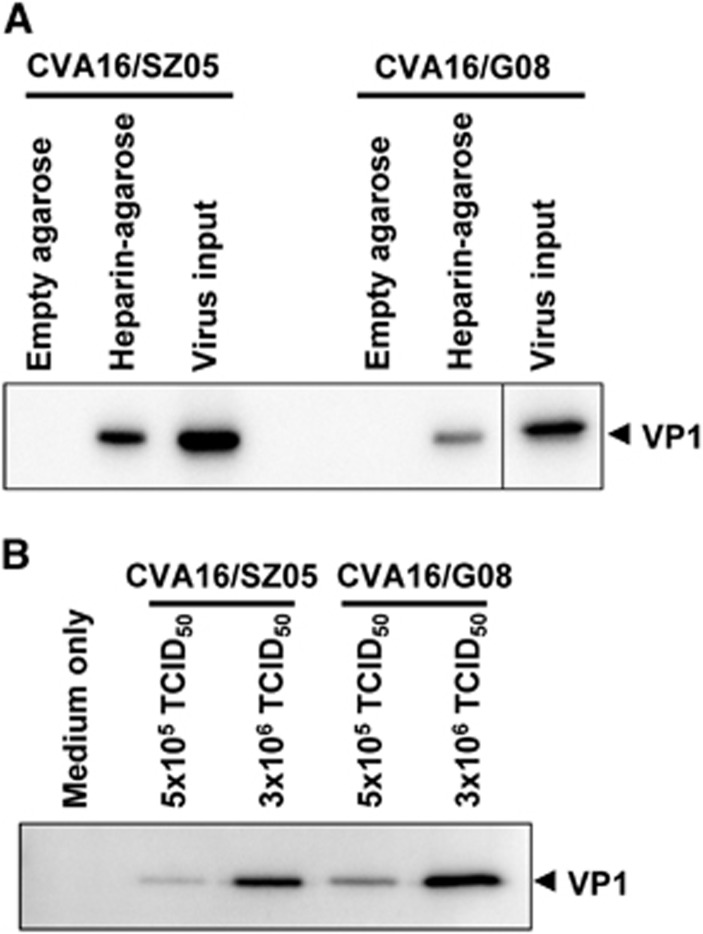
CVA16 directly interacts with heparin *in vitro*. (**A**) CVA16 specifically binds heparin-immobilized agarose beads. CVA16/SZ05 or CVA16/G08 (3 × 10^6^ TCID_50_ virus in a 500 μL final volume) was added to 20 μL of empty agarose beads or heparin-immobilized agarose beads, respectively, followed by incubation at 4 °C for 2 h to allow interaction. After separation by brief centrifugation, the precipitated agarose beads were washed with PBS, collected and analyzed for CVA16 protein by western blotting with an anti-VP1 polyclonal antibody. The virus input was also subjected to western blotting. (**B**) CVA16 binds heparin-immobilized agarose beads in a virus dose-dependent manner. Different amounts (5 × 10^5^ or 3 × 10^6^ TCID_50_) of CVA16/SZ05 or CVA16/G08 in a 500 μL final volume were mixed with 20 μL of heparin-immobilized agarose beads, followed by incubation at 4 °C for 2 h to allow interaction. Beads treated with medium-only served as a control in the assay. After incubation, the beads were processed and analyzed as described above.

To determine whether there is a direct interaction between CVA16 and heparan sulfate, we performed pull-down assays using heparin-immobilized agarose beads. Empty agarose beads without heparin conjugation were also used in the assays, serving as the negative control. As shown in Figure 4A, the presence of CVA16 VP1 protein was strongly detected in the samples pulled down by the heparin-immobilized agarose beads but not in those pulled down by the empty agarose beads, indicating that CVA16 specifically bound heparin–agarose. This specific binding was observed for both of the tested CVA16 strains, CVA16/SZ05 and CVA16/G08, suggesting that CVA16 binding to heparin–agarose is strain independent. In addition, the amount of pulled down CVA16 was dependent on the amount of virus input (Figure 4B), confirming the specificity of the interaction. These results clearly demonstrate that CVA16 is able to directly bind heparin *in vitro*.

### Mutation of surface-exposed positively charged residues of VP1 impairs CVA16 binding to heparin

**Figure 5:**
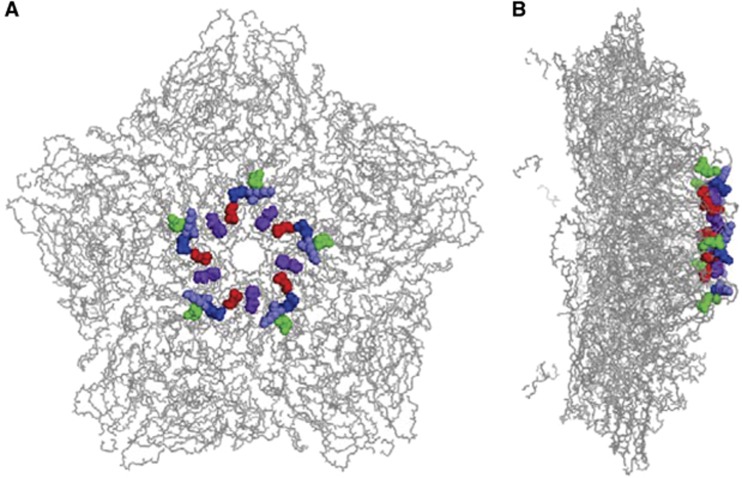
Location of five positively charged residues of VP1 in the CVA16 capsid. The five positively charged residues of VP1 are located in a cluster at the five-fold vertex of the CVA16 capsid. The three-dimensional structure of a pentamer of the CVA16 mature virion (PDB code: 5C4W)^14^ is presented with sticks. The five VP1 residues, K141, R166, K241, K242 and H245, are highlighted in red, green, blue, light blue and purple, respectively. (**A**) Top view of the CVA16 pentamer. (**B**) Side view of the CVA16 pentamer.

It has been postulated that positively charged residues symmetrically arranged in a cluster near the five-fold axis of enterovirus virions are responsible for their interaction with heparan sulfate.^24^ On the basis of the recently published crystal structure (PDB code: 5C4W) of a CVA16 mature virion,^14^ five positively charged residues (K141, R166, K241, K242 and H245) of VP1 are highly exposed on the surface of the CVA16 capsid, and they are located as clusters close to the five-fold axis (Figure 5).

**Figure 6:**
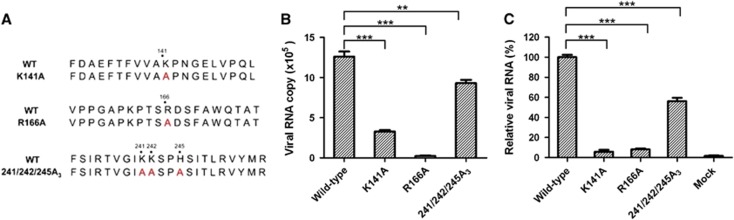
Effect of alanine substitution of the five positively charged residues of VP1 on heparan sulfate binding and cellular attachment of CVA16. (**A**) Sequence of the designed CVA16 mutants. (**B**) Analysis of heparin-binding ability of the CVA16 mutants by pull-down assays. Equal amounts (equivalent to 4 × 10^6^ copies of viral RNA genome) of CVA16 mutant or wild-type viruses were separately mixed with 20 μL heparin–agarose beads. The mixtures were incubated at 4 °C overnight. After three washes with PBS, the agarose beads were subjected to RNA extraction and qRT-PCR analysis as described in the ‘Materials and Methods’ section. The data are expressed as the mean±sd of the absolute viral RNA copies of triplicate samples. Representative results from two independent experiments are shown. (**C**) Ability of the mutant viruses to bind RD cells. The same amount of wild-type or mutant virus was added to RD cells and incubated at 4 °C for 1 h. Cell-bound virus was quantified by qRT-PCR. The data are reported as the mean±sd of the relative viral RNA genome numbers. Representative results from two independent experiments are shown. Statistical significance is indicated as follows: **P<0.01; ***P<0.001.

To assess the contributions of the five positively charged residues to the binding of CVA16 to heparan sulfate, three mutant forms of CVA16 were designed. The first and second mutants contained a single amino-acid replacement with alanine (A) at positions 141 and 166 of VP1, respectively (designated K141A and R166A, respectively). As K241, K242 and H245 are very close, the third mutant was designed to carry three simultaneous changes at positions 241, 242 and 245 (designated 241/242/245A_3_) (Figure 6A). These three CVA16 mutants were generated by using a reverse genetics system previously established.^13^ Rescued wild-type CVA16 was in parallel generated from a wild-type CVA16 cDNA clone,^13^ serving as the control in subsequent analyses. We should mention that the three CVA16 mutants are prone to reversion. Specifically, sequencing analysis revealed that mutant viruses collected at 28 h post transfection retained an RNA genome with the designed mutations, whereas reversion had occurred in viruses collected at 48 h post transfection (data not shown). Similarly, one passage of the mutant viruses collected at 28 h post transfection in fresh RD cells also resulted in the generation of revertants (data not shown). Therefore, only the viruses collected at 28 h post transfection were used in the following experiments.

We performed pull-down assays to determine the heparin-binding ability of the three CVA16 mutants. Heparin-bound viruses were quantified by determining viral RNA genome copies. As shown in Figure 6B, a significant decrease in the amount of pulled down viruses was detected for all three CVA16 mutants compared to the wild-type virus, with the greatest reduction (by 98%) observed for the R166A mutant. These results indicate that the heparin-binding ability of the three CVA16 mutants was significantly impaired.

### CVA16 mutants exhibit reduced attachment to target cells

We further evaluated the CVA16 mutants for their ability to bind target cells. As shown in Figure 6C, significantly lower amounts of cell-bound virus were detected in cells inoculated with the CVA16 mutants than in those inoculated with the wild-type virus, indicating that the attachment ability of the CVA16 mutants was impaired.

## DISCUSSION

The first step of viral entry is the attachment of viruses to the surface of permissive cells, which is usually mediated by a corresponding host receptor. Previously, SCARB2 was identified as a functional receptor for CVA16 as well as for EV71.^16, 17^ However, SCARB2 is a transmembrane protein predominantly expressed in endosomes and lysosomes^33^ and is barely present on the cell surface. The localization pattern of SCARB2 suggests that it is unlikely to mediate EV71/CVA16 binding on the cell surface; rather, it is involved in virus internalization and the subsequent uncoating process. Indeed, a recent report showed that SCARB2 does not play a significant role in EV71 attachment to susceptible cells.^34^ Therefore, SCARB2 is unlikely to be responsible for CVA16 attachment. In this study, we discovered that surface heparan sulfate serves as an attachment receptor for CVA16. Evidence that supports this finding includes: (i) pre-incubation with soluble heparin inhibited the attachment and infectivity of CVA16 (Figure 1); (ii) enzymatic removal of surface heparan sulfate reduced CVA16 attachment on permissive cells (Figure 2); (iii) blockade of heparan sulfate biosynthesis impaired the binding and infection by CVA16 (Figure 3); (iv) CVA16 directly interacted with heparin *in vitro* (Figure 4). These data convincingly demonstrate that cell surface heparan sulfate acts as a cellular receptor to facilitate CVA16 attachment to target cells. Previous studies have also shown that heparan sulfate GAGs on the cell surface serve as an attachment receptor for a number of picornaviruses such as echovirus,^19^ coxsackieviruses B3 and A9,^24, 25^ foot-and-mouth disease virus^26^ and enterovirus 71.^22^ These results, together with our own data, suggest that heparan sulfate-mediated viral attachment is likely a general mechanism involved in picornavirus entry.

It has been proposed that enteroviruses bind heparan sulfate via electrostatic interactions between negatively charged moieties of heparan sulfate and positively charged patches on the capsid surface.^22, 24^ For instance, Tan *et al.*^22^ predicted that clustering of three positively charged residues (R166, K242 and K244 of VP1) at the five-fold axis of EV71 capsids mediates heparan sulfate binding. A recent mutagenesis study showed that EV71 mutants with K242A and/or K244A mutation exhibited significantly reduced heparin binding and cell attachment, thereby demonstrating the importance in EV71 of VP1 residues K242 and K244 in its interaction with heparan sulfate.^35^ In the present study, we demonstrated that positively charged residues on the surface of CVA16 virions play an important role in heparan sulfate binding. We found that five positively charged residues (K141, R166, K241, K242 and H245) of VP1 were critical to the CVA16–heparan sulfate interaction, as single or combined substitution of the five residues with alanine resulted in the loss of heparan sulfate binding and viral attachment to different extents (Figure 6). Notably, these five residues are clustered near the five-fold axis (Figure 5), contributing to the formation of positively charged patches.^14^ In addition, sequence alignment reveals that four (K141, R166, K242 and H245) of the five residues are conserved among different CVA16 strains (data not shown). These findings strongly suggest symmetry-related clustering of positive charges as the mechanism by which CVA16 binds heparan sulfate. Among the five residues, R166 appeared to be the most important one, as replacement of this residue with alanine (R166A) resulted in nearly complete abolishment of heparan sulfate-binding activity and cellular attachment (Figure 6). We also noted that the three CVA16 mutants were prone to rapid reversion (data not shown), implying that the five positively charged VP1 residues play an important role in the CVA16 life cycle.

In summary, the present study revealed that CVA16 utilizes cell surface heparan sulfate to mediate viral attachment, and several positively charged residues critical to heparan sulfate binding and cellular attachment of CVA16 were identified. These findings provide insight into the early events of CVA16 entry, and may therefore facilitate rational design of anti-CVA16 drugs.
